# Correlation between standardized uptake value of ^18^F-FDG PET/CT and conductivity with pathologic prognostic factors in breast cancer

**DOI:** 10.1038/s41598-023-36958-9

**Published:** 2023-06-17

**Authors:** Dong-Joo Shin, Hongyoon Choi, Dong Kyu Oh, Hyun Pil Sung, Jun-Hyeong Kim, Dong-Hyun Kim, Soo-Yeon Kim

**Affiliations:** 1 Department of Radiology, Seoul National University Hospital, Seoul, Republic of Korea; 2 Department of Radiology, Seoul National University College of Medicine, 101 Daehak-ro, Jongno-gu, Seoul, 110-744 Republic of Korea; 3 Institute of Radiation Medicine, Seoul National University Medical Research Center, Seoul, Republic of Korea; 4 Department of Nuclear Medicine, Seoul National University Hospital, Seoul, Republic of Korea; 5 Department of Electrical and Electronic Engineering, Yonsei University, Seoul, Republic of Korea

**Keywords:** Breast cancer, Cancer imaging

## Abstract

We investigated the correlation between standardized uptake value (SUV) of ^18^F-fluorodeoxyglucose (FDG) positron emission tomography (PET)/computed tomography (CT) and conductivity parameters in breast cancer and explored the feasibility of conductivity as an imaging biomarker. Both SUV and conductivity have the potential to reflect the tumors’ heterogeneous characteristics, but their correlations have not been investigated until now. Forty four women diagnosed with breast cancer who underwent breast MRI and ^18^F-FDG PET/CT at the time of diagnosis were included. Among them, 17 women received neoadjuvant chemotherapy followed by surgery and 27 women underwent upfront surgery. For conductivity parameters, maximum and mean values of the tumor region-of-interests were examined. For SUV parameters, SUVmax, SUVmean, and SUVpeak of the tumor region-of-interests were examined. Correlations between conductivity and SUV were evaluated, and among them, the highest correlation was observed between mean conductivity and SUVpeak (Spearman’s correlation coefficient = 0.381). In a subgroup analysis for 27 women with upfront surgery, tumors with lymphovascular invasion (LVI) showed higher mean conductivity than those without LVI (median: 0.49 S/m vs 0.06 S/m, *p* < 0.001). In conclusion, our study shows a low positive correlation between SUVpeak and mean conductivity in breast cancer. Furthermore, conductivity showed a potential to noninvasively predict LVI status.

## Introduction

Electrical conductivity is one of the electric properties of tissues^1^. Previously, conductivity has long been investigated using the electrical impedance tomography systems^2^. The relatively recently introduced magnetic resonance electric properties tomography (MREPT) technique can reconstruct conductivity noninvasively by using the spin-echo based MRI sequences, without the need of an external electrode nor contrast injection^3,4^. The underlying biological mechanism for the elevated conductivity values is contributed to factors such as increased concentration and mobility of ions, increased tissue cellularity, and the breakdown of cell membrane^1,5–7^. According to an initial study on in vivo conductivity mapping of breast cancer using the MREPT technique, conductivity of fat, normal parenchyma, benign, and cancer were 0.07 S/m, 0.42 S/m, 0.56 S/m, and 0.89 S/m, respectively^8^.

Few MREPT studies on the diagnostic and prognostic values of conductivity in the field of breast imaging have been conducted. According to the initial study, conductivity values have shown the potential to differentiate malignant lesions from benign lesions, and invasive breast cancer from ductal carcinoma in situ^8^. In a study to evaluate the relationship between conductivity and apparent diffusion coefficient (ADC) of diffusion-weighted imaging, conductivity values have shown a negative correlation with ADC, but this correlation was abolished in the presence of necrosis^9^. The underlying mechanism of this observation is that conductivity is not affected by necrosis, whereas ADC is affected by necrosis^9^. Additionally, conductivity was associated with human epidermal growth factor receptor 2 (HER2) overexpression subtype of invasive breast cancer^10^. These results suggest the potential of conductivity parameters to differentiate the intrinsic subtypes of breast cancer^10^. However, a further study with larger sample size was required to confirm this association, and to identify the underlying mechanism for the association, since the number of tumors with HER2 overexpression was only eight in the study^10^. In summary, the previous MREPT studies in the field of breast imaging have demonstrated that conductivity likely reflects the heterogeneous nature of breast lesions as well as its potential as an imaging biomarker, although further studies are needed.

In contrast, the usefulness of ^18^F-fluorodeoxyglucose (FDG) positron emission tomography (PET)/computed tomography (CT) in the field of breast imaging is relatively well-known. ^18^F-FDG PET/CT has been used to evaluate tumor staging, treatment response, and monitoring recurrence and distant metastasis^11–14^. Multiple studies have demonstrated that the intensity of FDG uptake is associated with aggressiveness and prognostic factors of breast cancer, such as larger tumor size, high histologic grade, hormone receptor negativity, triple negativity, HER2 overexpression, and axillary lymph node (LN) metastasis^15–17^. The standardized uptake value (SUV) of ^18^F-FDG PET/CT is the most commonly used parameter to quantify the metabolic activity^14^. The maximum SUV (SUVmax) is the highest voxel value within the region-of-interest (ROI), and the mean SUV (SUVmean) is the mean value of all voxels within the ROI^14^. The peak SUV (SUVpeak) is the mean value within a 1 cm^3^ ROI surrounding the voxel with the highest activity^14^. Among these parameters, SUVmax has been most widely used in clinical practice, given its simplicity, reproducibility, and readily available software^14^. However, the main disadvantage of SUVmax is susceptible to image noise because the parameter represents a single-voxel value^14^. To overcome this problem, SUVpeak has been introduced^14^. The advantages of SUVpeak include being less sensitive to image noise compared to SUVmax while maintains the reproducibility^14^. The disadvantages of SUVpeak include reduced accuracy in the assessment of small lesions compared to SUVmax and the need for a specialized software^14^.

Although both conductivity and SUV have diagnostic and prognostic values in breast cancer, there have been no studies on the relationship between these two parameters. Based on these backgrounds, the purpose of this study was to investigate the correlation between conductivity and SUV, and compare their prognostic values by analyzing the relationship with clinicopathologic factors in breast cancer. In this study, for conductivity parameters, maximum and mean conductivity values of the tumor ROIs were evaluated, and for SUV parameters, SUVmax, SUVmean, and SUVpeak of the tumor ROIs were evaluated. One conductivity parameter and one SUV parameter with the maximum correlation level was selected, and the relationship between the imaging parameters and clinicopathologic factors were evaluated.

## Results

### Interobserver agreement of SUV and Conductivity

Interobserver agreement level was evaluated based on intraclass correlation coefficient (ICC). ICC of SUV parameters for the two readers was 0.991 in SUVmax, 0.987 in SUVmean, and 0.998 in SUVpeak, which is excellent level of concordance. ICC of conductivity parameters for the two readers was 0.601 in maximum conductivity, and 0.631 in mean conductivity, which is good level of concordance.

### Correlation between SUV and Conductivity

The relationship between conductivity (maximum and mean) and SUV (max, peak, and mean) was examined using the Spearman’s correlation coefficient (r) (Table 1). The correlation coefficients of maximum conductivity with SUVmax, SUVpeak, and SUVmean were 0.256, 0.298, and 0.237, respectively. As the correlation coefficients were ranged from 0.2 to 0.3, those were interpreted as negligible. The correlation coefficients of mean conductivity with SUVmax, SUVpeak, and SUVmean were 0.328, 0.381, and 0.307, respectively. As the correlation coefficients were ranged from 0.3 to 0.4, those were interpreted as low positive. Among all of the correlations, mean conductivity and SUVpeak showed the highest level of correlation (r = 0.381). Therefore, the two parameters (mean conductivity and SUVpeak) were selected for the subsequent analyses.

**Table 1:** Spearman correlation coefficient between SUV and conductivity parameters.

	SUVmax		SUVpeak		SUVmean	
	Spearman coefficient	*p* value	Spearman coefficient	*p* value	Spearman coefficient	*p* value
Maximum conductivity	0.256	0.094	0.298	0.049	0.237	0.122
Mean conductivity	0.328	0.03	0.381	0.011	0.307	0.043

### Association between clinicopathologic factors and SUVpeak

First, we examined univariable associations between clinicopathologic factors and SUVpeak using the simple linear regression analyses. As demonstrated in Table 2, tumor size 2 cm or larger, HER2-positive or triple negative subtype, and high level axillary LN metastasis were associated with higher SUVpeak values. Specifically, tumors 2 cm or larger had significantly higher median SUVpeak value than those smaller than 2 cm (median: 6.42 for tumors 2 cm or larger vs 2.47 for tumors smaller than 2 cm, *p* = 0.004). Tumors with HER2-positive or triple negative subtype had significantly higher median SUVpeak value than those with luminal subtype (median: 6.71 for HER2-positive or triple negative subtype vs 4.16 for luminal subtype, *p* = 0.039). Tumors with high level axillary LN metastasis had significantly higher median SUVpeak value than those with no or low level axillary LN metastasis (median: 8.59 for high level vs 4.52 for no or low level, *p* = 0.048). These data are also provided as a form of the box-and-whisker plots (Supplementary Fig. S1a–c).

**Table 2:** SUVpeak values according to clinicopathologic factors.

Variables	Median	IQR	*p* value*
Tumor size			0.004
< 2 cm	2.47	1.75, 4.52	
≥ 2 cm	6.42	4.73, 9.61	
ER			0.264
Negative	6.71	4.75, 10.54	
Positive	4.5	2.20, 7.79	
PR			0.902
Negative	4.8	3.00, 9.54	
Positive	4.74	2.19, 7.91	
HER2			0.071
Negative	4.51	2.2, 7.39	
Positive	6.42	4.49, 11.81	
Ki67			0.504
< 14%	4.63	2.23, 7.86	
≥ 14%	6.1	3.59, 10.87	
Tumor subtype			0.039
Luminal	4.16	2.18, 7.10	
HER2-positive or triple negative	6.71	4.55, 10.87	
Axillary LN metastasis level			0.048
No or low	4.52	2.23, 7.4	
High	8.59	5.17, 11.58	
Histologic grade			0.965
Low/intermediate	4.74	2.27, 8.34	
High	6.35	3.09, 9.22	

*ER* estrogen receptor, *HER2* human epidermal growth factor receptor type 2, *IQR* interquartile range, *LN* lymph node, *PR* progesterone receptor.

*p value obtained using simple linear regression analysis.

Next, we identified clinicopathologic factors independently associated with SUVpeak using the multiple linear regression analysis (Table 3). Among the three clinicopathological factors (tumor size, subtype, and axillary LN metastasis level), only the tumor size was independently associated with SUVpeak (*p* = 0.023). Tumor subtype and axillary LN metastasis level were not independently associated with SUVpeak in the multiple linear regression analysis (*p* = 0.294 and 0.104, respectively).

**Table 3:** Multiple linear regression analysis between associated factors and SUVpeak.

Variables	Beta coefficient	Standard error	*p* value
Tumor size	0.344	1.221	0.023
Tumor subtype	0.155	1.199	0.294
Axillary LN metastasis level	0.229	1.463	0.104

### Association between clinicopathologic factors and mean conductivity

Associations between clinicopathologic factors and mean conductivity were evaluated using the simple linear regression analyses, as provided in Table 4. Only tumor size was significantly associated with mean conductivity. Specifically, tumors 2 cm or larger showed higher median conductivity values than those smaller than 2 cm (median: 0.32S/m for tumors 2 cm or larger vs 0.16S/m for tumors smaller than 2 cm, *p* = 0.02) (Supplementary Fig. S1d).

**Table 4:** Mean conductivity values according to clinicopathologic factors.

Variables	Median	IQR	*p* value*
Tumor size			0.02
< 2 cm	0.16	− 0.03, 0.34	
≥ 2 cm	0.32	0.20, 0.49	
ER			0.852
Negative	0.29	0.21, 0.37	
Positive	0.29	0.06, 0.47	
PR			0.655
Negative	0.28	0.16, 0.38	
Positive	0.31	0.06, 0.50	
HER2			0.145
Negative	0.24	0.06, 0.38	
Positive	0.31	0.20, 0.41	
Ki67			0.78
< 14%	0.27	0.06, 0.45	
≥ 14%	0.31	0.19, 0.33	
Tumor subtype			0.104
Luminal	0.19	0.002, 0.46	
HER2-positive or triple negative	0.30	0.21, 0.40	
Axillary LN metastasis level			0.148
No or low	0.27	0.06, 0.38	
High	0.35	0.21, 0.54	
Histologic grade			0.438
Low/intermediate	0.30	0.10, 0.43	
High	0.23	0.02, 0.34	

*ER* estrogen receptor, *HER2* human epidermal growth factor receptor type 2, *IQR* interquartile range, *LN* lymph node, *PR* progesterone receptor.

*p value obtained using simple linear regression analysis.

### Association between postoperative pathologic factors and SUVpeak or mean conductivity

**Figure 1:**
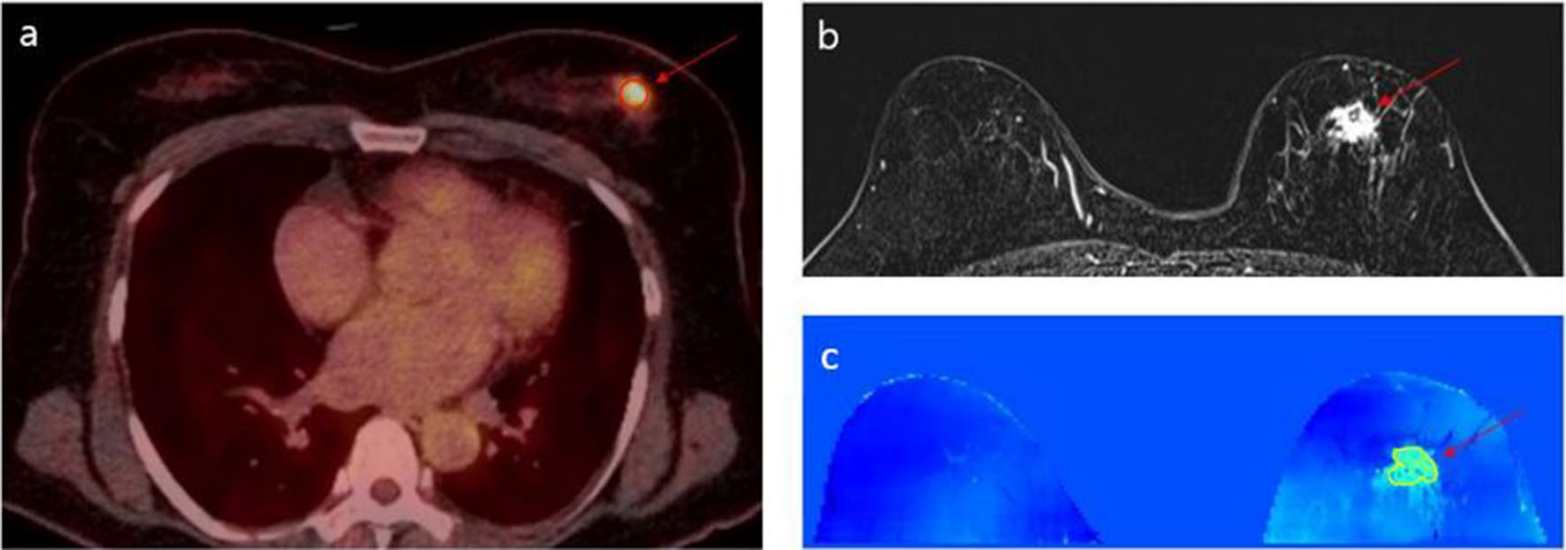
Images in a 55-year-old woman with a 3.2 cm human epidermal growth factor receptor type 2-positive invasive ductal carcinoma with lymphovascular invasion in left breast. (**a**) ^18^F-FDG PET/CT shows a high tumor SUVpeak value of 5.2 (arrow). (**b**) T1-weighted contrast-enhanced subtraction image shows an irregular heterogeneously enhancing mass (arrow). (**c**) Conductivity map shows a high tumor mean conductivity value of 0.33 S/m (arrow). Both SUV and conductivity measures showed high values in tumor with lymphovascular invasion.

**Figure 2:**
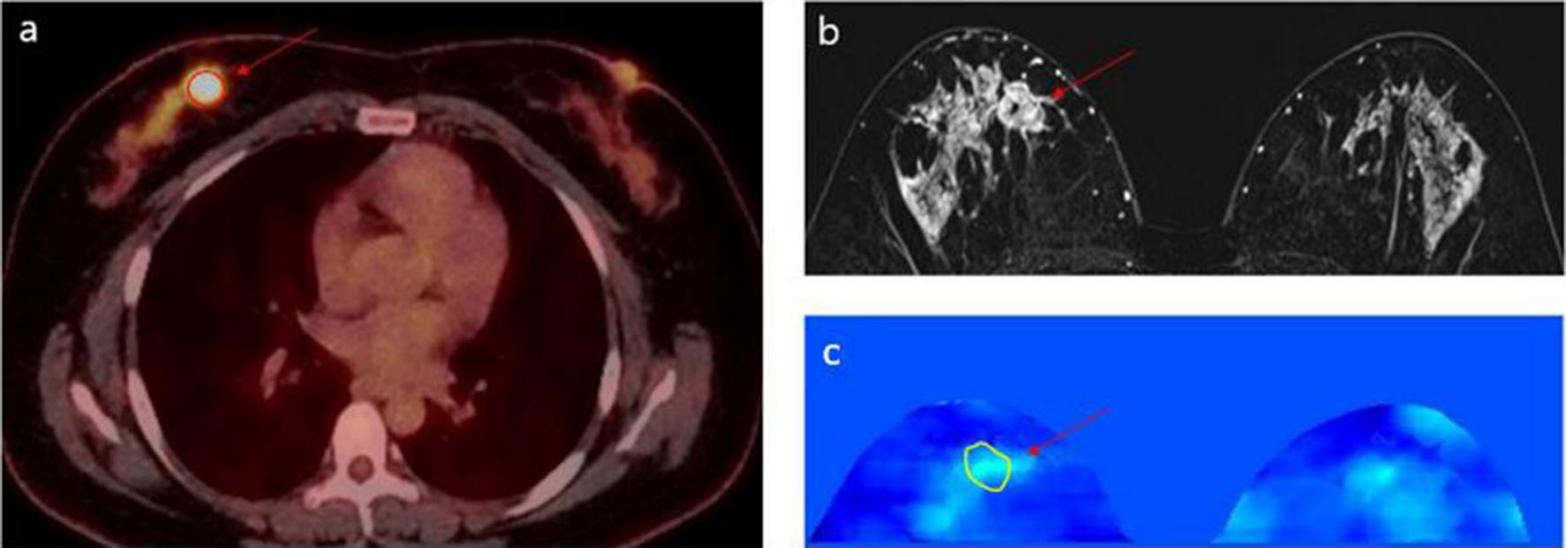
Images of a 41-year-old woman with a 2.2 cm luminal-type invasive ductal carcinoma without lymphovascular invasion in right breast. (**a**) ^18^F-FDG PET/CT shows a high tumor SUVpeak value of 9.13 (arrow). (**b**) T1-weighted contrast-enhanced subtraction image shows an irregular heterogeneously enhancing mass (arrow) in the marked background parenchymal enhancement. (**c**) Conductivity map shows a relatively low mean conductivity value of 0.06 S/m (arrow). While SUV showed high value despite the lack of lymphovascular invasion, mean conductivity showed relatively low value.

We performed a subgroup analysis with 27 patients who underwent upfront surgery without neoadjuvant chemotherapy (NAC) to evaluate the association between imaging parameter and postoperative pathologic factors (Table 5). Tumors with lymphovascular invasion (LVI) showed higher SUVpeak values than tumors without LVI, although the statistical significance was not reached (median: 2.29 for negative LVI vs 5.24 for positive LVI, *p* = 0.061) (Supplementary Fig. S1e). On the otherhand, tumors with LVI showed significantly higher mean conductivity values than those without LVI (median: 0.06S/m for negative LVI vs 0.49S/m for positive LVI, *p* < 0.001) (Supplementary Fig. S1f). Figures 1, 2 provide representative images with and without LVI, respectively.

**Table 5:** Mean conductivity and SUVpeak values according to surgical pathologic factors in women without neoadjuvant chemotherapy.

Variables	Mean conductivity		SUVpeak	
	Median (IQR)	*p* value	Median (IQR)	*p* value
LVI		< .001		0.061
Negative	0.06 (− 0.10, 0.31)		2.29 (1.86, 5.92)	
Positive	0.49 (0.34, 0.58)		5.24 (4.42, 7.50)	
Total tumor size		0.262		0.425
< 2 cm	0.11 (− 0.16, 0.32)		2.21 (1.86, 7.00)	
≥ 2 cm	0.32 (− 0.02, 0.47)		4.45 (2.22, 6.18)	
Invasive tumor size		0.067		0.07
< 2 cm	0.11 (− 0.08, 0.33)		2.29 (1.75, 5.22)	
≥ 2 cm	0.34 (0.06, 0.51)		4.85 (3.09, 7.10)	
LN metastasis		0.633		0.295
Yes	0.26 (− 0.08, 0.34)		3.39 (1.86, 5.28)	
No	0.26 (0.06, 0.49)		4.50 (2.21, 6.86)	
pN stage		0.576		0.584
pN0	0.26 (− 0.08, 0.34)		4.50 (2.22, 6.86)	
pN1	0.21 (0.06, 0.49)		3.31 (1.79, 5.53)	
pN2	0.37		3.39	

*IQR* interquartile range, *LN* lymph node, *LVI* lymphovascular invasion.

## Discussion

Conductivity, one of the electrical properties of tissues, can be reconstructed from MR images by the EPT technique^3,4^. Several studies have demonstrated that the conductivity values of breast cancers are higher than those of benign breast lesions or normal breast parenchyma, probably due to the increased concentration and mobility of ions, increased cellularity, and the breakdown of cell membrane in breast cancers^1,5–7^.

SUV of ^18^F-FDG PET/CT is the most commonly used parameter to quantify the metabolic activity of tissues^14^. It is known that high SUV is associated with aggressive tumor characteristics such as larger tumor size and non-luminal subtype^15–17^. SUVmax, SUVmean, and SUVpeak have been utilized in clinical practice, and among them, SUVmax has been most widely used given its simplicity, reproducibility, and readily available software^14^. However, SUVmax is susceptible to image noise because the parameter represents a single-voxel value^14^. To overcome this problem, SUVpeak has been introduced^14^. SUVpeak is less sensitive to image noise while maintains reproducibility^14^.

Both conductivity and SUV have the potential to reflect the heterogeneous tumor characteristics, but the relationships between the two parameters have not been investigated until now. Therefore, in this study, we investigated the correlation between conductivity and SUV. For conductivity parameters, maximum and mean conductivity value of the tumor ROI were examined. For SUV parameters, SUVmax, SUVmean, and SUVpeak of the tumor ROI were examined. Our study found that conductivity and SUV generally showed positive correlations although the correlation level was low. Among the correlations of the parameters, mean conductivity and SUVpeak showed the highest level of correlation. Besides, similar to the aforementioned advantages of SUVpeak, mean conductivity can be less sensitive to image noise than maximum conductivity, as the maximum conductivity represents a single-voxel value. Therefore, we decided to focus on the mean conductivity and SUVpeak when analyzing the associations of the imaging parameters with clinicopathologic factors.

The positive correlation between conductivity and SUV is supported by the known observations as follows. Both SUV and conductivity show higher values in malignant breast lesions compared to benign lesions^5,18^. High SUV values are associated with increased glycolysis and high cellularity of tumors^18–21^. Malignant breast lesions tend to show higher conductivity values than benign lesions, due to the increased sodium concentration and water content, increased membrane permeability, and decreased membrane potential in malignant breast lesions^22^. Of note, it should be mentioned that the level of correlation between the two parameters is low. The small sample size of this study may prevent the precise measurement of correlation.

In terms of the associations of SUVpeak with clinicopathologic factors, SUVpeak was associated with tumor size, axillary LN metastasis level, and tumor subtype on the simple linear regression analysis. That is, tumors 2 cm or larger, high LN metastasis level, and HER2 positive or triple-negative subtype were associated with higher SUVpeak, compared to their counterparts. Among the three clinicopathologic factors, the tumor size alone was independently associated with SUVpeak on the multiple linear regression analysis. Our findings were consistent with previous literature, suggesting that SUV reflects metabolic aggressiveness of breast cancer^19,23^. However, caution is needed when interpreting our results due to the small sample size per each clinicopathologic factors. Tumors smaller than 1 cm in diameter are not reliable to measure SUV, because of partial volume effect and limited spatial resolution of FDG-PET^18^. However, only one patient had tumor size less than 1 cm in our study, thus the partial volume effect might be minimal.

Regarding the associations of mean conductivity with clinicopathologic factors, mean conductivity showed a significant association with tumor size as well. Tumors 2 cm or larger showed higher conductivity values than those smaller than 2 cm. A possible explanation is that large tumors tend to have high cellularity, considering their expansile and aggressive nature. High cellularity increases ion concentration and eventually leads to high conductivity^1,5–7^. However, there are conflicting results regarding our findings. While Kim et al^9^ demonstrated similar results, Shin et al.^8^ showed no significant association between tumor size and conductivity in breast cancer. Hence, future research with a larger sample size might be needed for further investigation.

Conductivity value is mainly affected by ion concentration and mobility in tissue components^1^. Moreover, ion concentration is independent of frequency, but ion mobility changes according to frequency level^1^. Cell membrane works as an insulator in low frequencies, thus electrical impedance increases. Conversely, it works as a conductor in high frequencies, thus electrical impedance decreases^22^. In particular, frequencies over 100 MHz are known to effectively differentiate pathologic from normal tissues^24^. Frequency of 3.0-T MRI used in this study is approximately 127 MHz, which is within the suggested level.

In our subgroup analysis for patients who underwent upfront surgery without NAC, positive LVI status in the surgical specimen was significantly associated with higher mean conductivity value. In contrast, there was no statistical significance between SUVpeak and LVI status. LVI is defined as the presence of tumor emboli in lymphatics or blood vessels in tumor bed^25^. It is associated with a higher risk of LN and systemic metastasis, and worse survival outcome in breast cancer^26,27^. Thus, there is an increasing need to evaluate LVI status noninvasively and quantitatively to predict prognosis and to monitor response of anti-lymphangiogenic therapies in breast cancer^28^.

Possible explanations for association between LVI and conductivity are as follows. First, LVI is associated with high cellularity and cell proliferation in breast cancer^29,30^. Conductivity is also associated with high cellularity, as evidenced by a negative correlation with ADC^9^. High cellularity leads to high ion concentration, thus conductivity may increase in tumors with LVI^1^. Second, when tumor cells invade lymphatic vessels, they disrupt the basement membrane and lymphovascular endothelial wall, inducing increased permeability^31^. Since cell membrane is composed of lipids, it acts as insulator of electric currents^7^. Therefore, when cell membrane is disrupted, its insulating property is lost, and conductivity may increase.

In contrast, in a previous study of Kim et al.^10^, breast cancers with LVI showed lower conductivity values than those without LVI in tumors 2 cm or larger. However, the association was not significant in tumors smaller than 2 cm, and LVI was not independently associated with conductivity after adjusting for confounding factors in multivariate analysis. On the otherhand, in our study, conductivity still showed an independent association with LVI, even after adjusting for the influence of tumor size in multilinear regression analysis. Further study with a larger sample size might be needed to confirm the relationship.

### Limitations

There are several limitations in our research. First, it is a retrospective single-center study, so there might be a selection bias. Second, only 44 women were investigated in this study. This small sample size is one of the limitations of our study which limits the valid conclusion and precise evaluation. The purpose of this study was to evaluate the correlation between conductivity and SUV of PET-CT, therefore, only women who underwent both breast MRI containing non-fat-suppressed T2-weighted TSE sequence for conductivity study and PET-CT were able to be included. The small sample size of this study may prevent the precise measurement of correlation between SUV and conductivity values. The low-level of correlation between the two parameters may be associated with our small sample size. Further study with a larger sample size and external dataset will be needed to conclude the relationship between the two parameters. Third, this study did not have a control group. All included women had breast cancers, and we evaluated the conductivity and SUV values of the tumor ROI. We did not evaluate the conductivity and SUV values for benign breast lesions and normal breast tissues. It is known that conductivity values of normal breast tissue and benign breast lesions are generally lower than those of breast cancers, but both false-negative and false-positive conductivity patterns may be possible due to the limitations of the phase-based MREPT technique used in this study (e.g. low SNR, spatial heterogeneity of the magnetic field, low resolution, and chemical shift artifacts)^5,8^. The presence of a control group could have provided more objective and diverse information to our study. Fourth, ICC of conductivity value was lower than that of SUV value. Hence, the reliability of conductivity measurements should be improved in future studies. Lastly, the phase-based MREPT technique used in this study has technical issues with spatial heterogeneity of the transmit/receive magnetic fields and low SNR. We tried to overcome these issues by using the coil combination technique, the use of an optimal kernel size, and the weighted polynomial fitting technique^5,32^. However, the precision of the conductivity measurements and the validity of the study results may have been hampered by these restrictions. Recently introduced deep-learning based reconstruction technique may improve the SNR compared to the current phase-based MREPT technique^33^. Further technical developments and application will be needed.

## Conclusion

In conclusion, this study found a low positive correlation between SUVpeak and mean conductivity. Tumor size was associated with SUVpeak and mean conductivity, with larger SUVpeak and conductivity values ​​for tumors 2 cm or larger than those smaller than 2 cm. Furthermore, conductivity showed a potential to noninvasively predict LVI status of breast cancers.

## Materials and methods

This retrospective single-center study was approved by the Institutional Review Board of Seoul National University Hospital (No. 2110–134-1264). The requirement for informed consent was waived by the IRB, because of the retrospective study design. All methods were conducted in accordance with the Declaration of Helsinki.

### Patients

Among the patients who underwent breast MRI in our institution between July 2017 and January 2018, patients with following criteria were included in the study. The inclusion criteria were (1) patients who were newly diagnosed as breast cancer by percutaneous core needle biopsy, (2) patients who underwent both breast MRI and PET/CT for tumor staging, and (3) patients with breast MRI containing non-fat-suppressed T2-weighted turbo-spin-echo (TSE) sequence for conductivity study. A total of 44 women were included in this study; 27 and 17 underwent upfront surgery and preoperative NAC, respectively.

### Patient characteristics

A total of 44 women were analyzed, and the clinicopathologic characteristics are described in Table 6. The median patient age was 54 years with ranges from 32 to 77 years. Most women had invasive ductal carcinoma with low or intermediate histologic grade. Twenty four (55%) women had luminal subtype, 15 (34%) had HER-2 positive, and 5 (11%) had triple negative subtype. In addition, 27 (61%) women had tumors sized ≥ 2 cm measured on pretreatment breast MRI. Sixteen (36%) women had LN metastasis, and 12 (27%) had LVI. Seventeen (39%) women received NAC preoperatively, and 27 (61%) underwent upfront surgery. Women with NAC had larger tumor size on MRI than those without NAC (median: 1.8 cm vs 3 cm, *p* = 0.003). Additionally, women with NAC had triple-negative or HER2-positive breast cancers (71% [12 of 17]) more frequently than those without NAC (26% [7 of 27], *p* = 0.001).

**Table 6:** The characteristics of the patients included in the study.

Characteristics	Values	Frequency (%)
Age at diagnosis (years)	Median: 54 (ranges, 32–77)	
Histology		
Invasive ductal carcinoma	39	89
Invasive lobular carcinoma	4	9
Mucinous carcinoma	1	2
Histological grade		
Low or intermediate	34	77
High	10	23
ER positive	32	73
PR positive	23	52
ER or PR positive	33	75
HER2 positive	15	34
Tumor size		
< 2 cm	17	39
≥ 2 cm	27	61
Axillary LN metastasis level		
No metastasis	22	50
Low level (I)	14	32
High level (II-III)	8	18
Subtype		
Luminal	24	55
HER2 positive	15	34
Triple negative	5	11
Neoadjuvant chemotherapy		
No	27	61
Yes	17	39
Pathologic nodal stage		
N0	28	64
N1	13	30
N2	3	7
Lymphovascular invasion		
No	32	73
Yes	12	27

*ER* estrogen receptor, *HER2* human epidermal growth factor receptor type 2, *LN* lymph node, *PR* progesterone receptor.

### Clinical and pathologic data collection

Prior to clinicopathologic analysis, data of tumor size, histologic type, histologic grade, estrogen receptor (ER), progesterone receptor (PR), HER-2, Ki-67, axillary LN metastasis level, LN burden, LVI status of each patient were collected. For patients who have not received NAC, information was collected based on pathologic report after operation. For patients who have received NAC, information was collected based on pathologic report of biopsy specimen. Data were obtained from different sources because after NAC, the characteristic of tumor and LVI status might change in the operation report compared to the initial biopsy report.

Radiologic tumor size was assessed in contrast-enhanced breast MRI, and was divided into groups of ≥ 2 cm or < 2 cm. The histological grade was divided into two groups for analysis; low to intermediate, and high grade. ER and PR positivity were defined as ≥ 1% tumor cells with nuclear staining using standard immunohistochemistry methods. HER-2 positivity was defined as immunohistochemistry HER-2 score of 3 + , or gene amplification by fluorescence in situ hybridization in tumors with HER-2 score of 2 + . Ki-67 was scored as the percentage of cells with positively stained nuclei in total tumor cells. Tumors with Ki-67 ≥ 14% were considered to have high proliferative activity^34^. Tumors were categorized into three subtypes based on receptor status^35^: (1) Luminal type (ER and/or PR positive, HER-2 negative), (2) HER-2 positive (regardless of ER and PR status), and (3) triple negative (ER, PR and HER-2 negative).

Axillary LN metastasis level was divided into low (I) or high (II-III) level. Axillary level was determined in surgical reports in women without NAC, while it was assessed based on pretreatment PET/CT or US in women with NAC. Postoperative pathologic data were obtained only in women without NAC, which includes pathologic total tumor size (both invasive and in situ components), invasive tumor size, LVI status, and pathological nodal stage (pN stage).

### MRI protocol

Breast MRI images were acquired with patients in the prone position using a 3.0-T scanner (MAGNETOM Skyra, Siemens Healthineers, *Erlangen, Germany*) with an 18-channel breast coil. The routine protocol consists of (1) an axial fat-suppressed T2-weighted spectral adiabatic inversion recovery TSE sequence, (2) dynamic contrast-enhanced (DCE)-MRI with an axial 3D fat-suppressed T1-weighted spoiled gradient-echo sequence before and five times after an intravenous bolus injection of 0.1 mmol/kg gadobutrol (Gadovist; Bayer), and (3) diffusion-weighted imaging (DWI) with a readout-segmented echo-planar imaging sequence (RESOLVE). In addition, axial noncontrast-enhanced non-fat-suppressed T2-weighted TSE sequence (repetition time/echo time: 9150/105 ms, flip angle: 140°, FOV: 320 × 320 mm, matrix: 384 × 260 pixels, slice thickness: 3 mm, acquisition time: 2 min 30 s) was acquired as raw data for conductivity reconstruction.

### Conductivity reconstruction

Conductivity reconstruction was performed using a phase-based MREPT with a multi-receiver coil combination technique, as described in detail previously^5,8,32^. Conductivity can be reconstructed as $\frac{{\nabla^{2} \varphi }}{2\mu 0\omega }$ ; where (1) φ is the phase of the transmit/receive magnetic radio-frequency field, (2) μ0 is the permeability of free space, and (3) ω is the Larmor frequency^36^. In this study, the phase of radiofrequency field was acquired based on non-fat-suppressed T2-weighted TSE sequence. Non-fat-suppressed image was used because fat region contributes to multi-receiver coil combination process as a homogeneous ROI, thus improving image quality with fewer artifacts^5^.

Spatial heterogeneity of transmit/receive magnetic field and low SNR are known concerns related to phase-based MREPT. First, spatial heterogeneity of received magnetic fields increases in breast MRI using multiple channel coils, which can eventually cause errors in conductivity estimation. Therefore, the coil combination technique was used to reduce the spatial heterogeneity of multiple channel coils^32^. Second, MREPT has a relatively low SNR owing to noise amplification. However, SNR can be improved by increasing the kernel size, although this results in a decreased image resolution. Therefore, a 31 × 31 two-dimensional (2D) kernel was used, which was chosen considering balance and trade-offs between SNR and image resolution^5^. Afterwards, weighted polynomial fitting technique was applied to compensate for image blurring^32^. One of the authors (J.H.K) reconstructed the conductivity map using MATLAB R2017b (Mathworks, Natick, MA, USA).

### PET/CT protocol

Whole body PET images were acquired using dedicated PET/CT scanners (Biograph 40 or Biograph 64, Siemens Healthcare, Knoxvillle, TN, USA) 1 h after intravenous injection of ^18^F-FDG with a radioactivity of 5.18 MBq/kg of body weight. All patients fasted for at least 6 h before tracer injection. The PET/CT acquisition protocol at our institution has been previously described in detail^37^. Briefly, CT images were obtained first, and PET images were obtained subsequently. CT was performed from skull base to the mid-thigh region, with 5 mm thickness at 50 mAs and 120 kVp. Then, PET scan was performed with 2 min/bed scan duration from mid-thigh to skull base. PET data were reconstructed with an ordered subset expectation maximization with 2 iterations and 21 subsets.

### Image analysis

For conductivity analysis, ROIs were drawn on non-fat-suppressed T2-weighted TSE axial image using MATLAB R2011a (Mathworks, Natick, MA, USA), with reference to DCE-MRI. Out of 50 slices of T2-weighted TSE images, slices that cover the whole tumor were selected. Two independent radiologists (D.J.S and S.Y.K with 2 and 10 years of experience, respectively) manually drew ROIs fitting to the tumors on the selected slices of T2-weighted TSE sequence. They were blinded to the clinicopathologic information of the tumor while measuring conductivity. Conductivity was calculated on each pixel, and the mean and maximum values were recorded.

A dedicated software package (Syngo.via, Siemens Medical Solutions, Erlangen, Germany) was used to analyze PET images. Two independent nuclear medicine physicians (H.C and D.K.O with 11 and 5 year of experience, respectively), blinded to the clinicopathologic information of the tumor, measured SUV values. The maximum (SUVmax), mean (SUVmean), and peak (SUVpeak) SUV were calculated in ROIs manually drawn by visual inspection on the area of the breast tumor containing the highest SUV pixel^14^. SUVmax is defined as the highest voxel value within the ROI, while SUVpeak is the mean value of radiotracer uptake within the ROI surrounding the pixel with the highest activity^14^. SUVmean is the mean value of all voxels within the ROI^14^.

### Statistical analysis

Tumor size and subtype were compared between women with and without NAC using Mann–Whitney U or chi-squared tests. Interobserver variability of conductivity and SUV was assessed using the ICC: poor (< 0.20), fair (0.21–0.40), moderate (0.41–0.60), good (0.61–0.80), or excellent (0.81–1.00)^38^.

The relationship between conductivity and SUV was examined using Spearman’s correlation coefficient. For conductivity parameter, maximum and mean conductivity of the tumor ROI were evaluated. For SUV parameter, SUVmax, SUVmean, and SUVpeak of the tumor ROI were evaluated. Therefore, the number of the combinations for the relationship between the two conductivity values and the three SUV values was six. Spearman’s correlation coefficient was defined as negligible (0–0.3), low positive (0.3–0.5), moderately positive (0.5–0.7), high positive (0.7–0.9), and very high positive (0.9–1.0)^39^. After evaluating the correlation of the two parameters, the mean conductivity and the SUVpeak were selected as a representative parameter for the subsequent analyses, because the two parameters showed the highest level of correlation.

Association between SUVpeak and clinicopathological factors were evaluated using the simple and multiple linear regression analyses. First, the simple linear regression analysis was performed to identify an univariable association between SUVpeak and clinicopathological factors. Next, the multiple linear regression analysis was performed to identify an independent association between SUVpeak and clinicopathological factors, using the variables (i.e., tumor size, tumor subtype, and axillary LN metastasis level) with p values < 0.05 on the simple linear regression analysis.

Association of mean conductivity with clinicopathological factors were evaluated using the simple linear regression analysis. The multiple linear regression analysis was not performed for mean conductivity, because only one clinicopathologic factor (i.e., tumor size) was associated with mean conductivity on the simple linear regression analysis.

Lastly, we performed a subgroup analysis with 27 patients who underwent upfront surgery without NAC to evaluate the association between imaging parameters (here, mean conductivity and SUVpeak) and postoperative pathologic factors. Simple linear regression analysis was performed to evaluate the association between imaging parameters and postoperative pathologic factors. All analyses were performed using SPSS software (PASW Statistics, version 20; SPSS, Chicago, IL, USA), and *p* values < 0.05 were considered statistically significant.

## Supplementary Information

Supplementary Figure S1.

## Data Availability

The datasets generated and analyzed during the current study are available from the corresponding author on reasonable request.
