# Supplementary material for: Correlation between standardized uptake value of 18F-FDG PET/CT and conductivity with pathologic prognostic factors in breast cancer
Source: Sci Rep. 2023 Jun 17;13:9844. doi: 10.1038/s41598-023-36958-9 (PMC10276807; doi:10.1038/s41598-023-36958-9)
Supplement: Supplementary file 1 — Supplementary Figure S1. [file 41598_2023_36958_MOESM1_ESM.docx]

**Supplementary Information**

**Supplementary Figure S1.** Box-and-whisker plots of SUVpeak and mean conductivity according to the clinicopathologic factors.


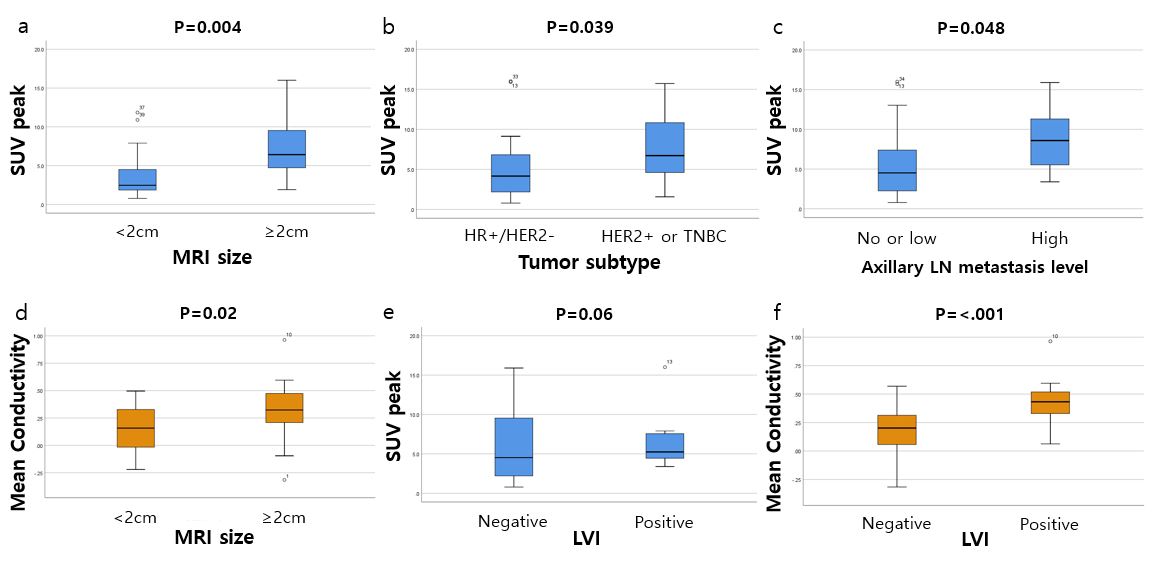


Note: HR, hormone receptor; HER2, human epidermal growth factor receptor type 2; TNBC, triple negative breast cancer; LVI, lymphovascular invasion
